# Humoral immune responses during SARS-CoV-2 mRNA vaccine administration in seropositive and seronegative individuals

**DOI:** 10.1186/s12916-021-02055-9

**Published:** 2021-07-26

**Authors:** Elizabeth Fraley, Cas LeMaster, Eric Geanes, Dithi Banerjee, Santosh Khanal, Elin Grundberg, Rangaraj Selvarangan, Todd Bradley

**Affiliations:** 1 Genomic Medicine Center, Children’s Mercy Research Institute, Children’s Mercy Kansas City, Kansas City, MO 64108 USA; 2 Department of Pathology and Laboratory Medicine, Children’s Mercy Kansas City, Kansas City, MO 64108 USA; 3 Department of Pediatrics, Children’s Mercy Kansas City, Kansas City, MO 64108 USA; 4 Department of Pediatrics, UMKC School of Medicine, Kansas City, MO 64108 USA; 5 Departments of Pediatrics and Pathology and Laboratory Medicine, University of Kansas Medical Center, Kansas City, KS USA

**Keywords:** SARS-CoV-2, Antibody response, mRNA vaccine

## Abstract

**Background:**

The global pandemic of coronavirus disease 2019 (COVID-19) is caused by infection with the SARS-CoV-2 virus. Currently, there are three approved vaccines against SARS-CoV-2 in the USA, including two based on messenger RNA (mRNA) technology that has demonstrated high vaccine efficacy. We sought to characterize humoral immune responses, at high resolution, during immunization with the BNT162b2 (Pfizer-BioNTech) vaccine in individuals with or without prior history of natural SARS-CoV-2 infection.

**Methods:**

We determined antibody responses after each dose of the BNT162b2 SARS-CoV-2 vaccine in individuals who had no prior history of SARS-CoV-2 infection (seronegative) and individuals that had previous viral infection 30–60 days prior to first vaccination (seropositive). To do this, we used both an antibody isotype-specific multiplexed bead-based binding assays targeting multiple SARS-CoV-2 viral protein antigens and an assay that identified potential SARS-CoV-2 neutralizing antibody levels. Moreover, we mapped antibody epitope specificity after immunization using SARS-CoV-2 spike protein peptide arrays.

**Results:**

Antibody levels were significantly higher after a single dose in seropositive individuals compared to seronegative individuals and were comparable to levels observed in seronegative individuals after two doses. While IgG was boosted by vaccination for both seronegative and seropositive individuals, only seronegative individuals had increased IgA or IgM antibody titers after primary immunization. We identified immunodominant peptides targeted on both SARS-CoV-2 spike S1 and S2 subunits after vaccination.

**Conclusion:**

These findings demonstrated the antibody responses to SARS-CoV-2 immunization in seropositive and seronegative individuals and provide support for the concept of using prior infection history as a guide for the consideration of future vaccination regimens. Moreover, we identified key epitopes on the SARS-CoV-2 spike protein that are targeted by antibodies after vaccination that could guide future vaccine and immune correlate development.

**Supplementary Information:**

The online version contains supplementary material available at 10.1186/s12916-021-02055-9.

## Background

Severe acute respiratory syndrome coronavirus 2 (SARS-CoV-2) is a novel betacoronavirus causing coronavirus disease 2019 (COVID-19) [1, 2]. Humoral immune responses play critical roles in protecting individuals against SARS-CoV-2 infection, particularly through the elicitation of neutralizing antibodies. There is an urgent need to understand humoral immune responses to SARS-CoV-2 and how these responses contribute to disease severity and vaccine-induced immunity. Early antibody responses targeting the SARS-CoV-2 spike protein (S) or nucleocapsid protein (NP) are detectable soon after natural infection, within 20 days of symptom onset, and have been demonstrated to be immunoglobulin M (IgM), IgG, and IgA isotypes with varying kinetics of development [3–7]. While IgG and IgM antibody responses have been more extensively studied in SARS-CoV-2 infection, there have been reports that IgA antibodies contribute to the early neutralizing antibody response [8]. Antibodies that can neutralize SARS-CoV-2 and prevent infection are targeted for therapeutics and vaccine development [9, 10].

There are two currently US Food and Drug Administration (FDA)-approved SARS-CoV-2 vaccines that are based on messenger RNA (mRNA) platform technology in the United States (US) and have demonstrated greater than 90% efficacy after two doses in phase III clinical trials (BNT162b2/Pfizer; mRNA-1273/Moderna) [11, 12]). The phase III trials of the mRNA vaccines primarily studied the immune responses in individuals who had no prior history of SARS-CoV-2 infection. Although immune correlates of protection for SARS-CoV-2 vaccines have not yet been defined in humans, animal studies with similar vaccine formulations have identified levels of neutralizing antibodies as one potential correlate of protective efficacy in rhesus macaques [13, 14]. With over 31 million cases of COVID-19 that have been documented in the US and high observed seroprevalence [15], it is critical to define the immune responses after vaccination in individuals with previous infection. We and others have demonstrated that after a single dose of SARS-CoV-2 mRNA vaccine, individuals with previous infection have more robust antibody responses when compared to infection-naïve individuals [16–19]. This boost of preexisting antibody immunity from prior infection may be considered when deciding whether a single or double dose is required for vaccine-mediated protection in individuals with prior history of infection.

Here, we report antibody responses in seronegative and seropositive healthcare workers at baseline and after each of the two doses of the BNT162b2 SARS-CoV-2 vaccine. We determined the levels of antibodies of multiple immunoglobulin subclasses to different viral antigens, identified potential SARS-CoV-2 neutralizing antibody levels, and identified key immunodominant peptides that are targeted on the SARS-CoV-2 spike protein after immunization.

## Methods

### Individuals and sample collection

We enrolled healthcare workers from our children’s hospital prior to the administration of the Pfizer BNT162b2 COVID-19 vaccine. Peripheral blood was collected before vaccination at baseline (week 0), after primary immunization (week 3), and secondary booster immunization (week 7) from healthcare workers who had no known history of infection (*N* = 152) or previous laboratory-confirmed SARS-CoV-2 infection 30–60 days (*N* = 42) prior to the administration of the BNT162b2 vaccine (administered at week 0 and week 3). This population consisted of mostly adult middle aged, white, females who did not identify as Hispanic or Latino (Supplementary Table 1). The SARS-CoV-2 vaccine specimens were collected at Children’s Mercy Kansas City and were reviewed and approved by the Children’s Mercy IRB. Serum or plasma was utilized to perform the immunoassays that were isolated from venous whole blood collection and stored in frozen in ultra-low temperature freezers until use.

### SARS-CoV-2 viral antigen multiplexed binding assay

To measure antibody levels to SARS-CoV-2 spike subunit proteins (spike subunit 1 (S1), spike subunit 2 (S2), receptor-binding domain (RBD)) and nucleocapsid protein (NP) antigens, we utilized a bead-based multiplex assay based on the Luminex xMAP technology using reagent kits that had secondary antibodies that were specific for immunoglobulin isotypes (IgG, IgM, IgA). We used the following kits: IgG (Millipore, #HC19SERG1-85 K), IgM (Millipore, #HC19SERM1-85 K), and IgA (Millipore, #HC19SERA1-85 K) following standard manufacture protocols. Each kit provided the same sets of SARS-CoV-2 antigen conjugated beads (S1, S2, RBD, NP) along with 4 positive control beads and a negative control bead set. The positive control beads were beads coated with different concentrations of IgG, IgM, or IgA (depending on the isotype kit utilized). The negative control beads did not have antigen conjugated to determine nonspecific binding. The 4 antigen-conjugated beads, 4 positive control beads, and 1 negative control beads were mixed and incubated with each plasma sample that was diluted 1:100 with assay buffer. With each assay plate, at least two sample wells with only buffer and no plasma were included to determine assay background. Finally, PE-anti-human IgG, IgM, or IgA conjugate detection antibodies were utilized to determine antibody isotype responses to each of the SARS-CoV-2 antigens. Using the positive control beads, we determined the inter-assay (plate-to-plate) coefficient of variation (CV) for each assay. We determined that the CVs were 5.16%, 7.42%, and 11.45% for the IgG, IgM, and IgA assays, respectively. In order to acquire and analyze data, we utilized the Luminex analyzer (MAGPIX) and Luminex xPONENT acquisition software. Samples were run in technical duplicate and after acquisition Net MFI was utilized which is MFI with background well (no plasma) MFI subtracted. Positive control beads were utilized to ensure positive detection of the well and to identify any inter- and/or intra-assay technical variation. We next determined the level of nonspecific binding by using the negative control samples MFI (beads without antigen mixed with plasma) for each isotype (IgG, IgM, IgA). The IgG had the highest background MFI, so we used the average MFI plus the standard deviation of the IgG samples to set the detection threshold for IgG, IgM, and IgA isotype assays (442 MFI).

### SARS-CoV-2 viral neutralizing antibody assays

To detect viral neutralizing antibodies the SARS-CoV-2 Surrogate Virus Neutralization Test kit was utilized (Genscript, #L00847) according to the standard protocol. Samples were run in duplicate with blocking values averaged. This kit detects antibodies that can block the interaction between the receptor-binding domain of the viral spike glycoprotein with the angiotensin-converting enzyme 2 (ACE2) cell surface receptor and has been approved by the FDA for emergency use. Plasma samples along with positive (anti-RBD antibody) and negative (buffer only) were incubated with a Horseradish peroxidase (HRP) conjugated recombinant SARS-CoV-2 RBD fragment. The mixture was then added to a capture plate that was coated with the human ACE2 protein. The unbound HRP-RBD will bind to the plate. After washing, 3,3′,5,5′-tetramethylbenzidine (TMB) solution was added to develop the HRP signal and was read at 450 nm in a microtiter plate reader. The absorbance of the sample is inversely dependent on the titer of the anti-SARS-CoV-2 neutralizing antibodies. Inhibition was calculated by (1 − OD value of sample/OD value of negative control) × 100 which gives percent inhibition. A cutoff of ≥ 30% is considered positive for SARS-CoV-2 neutralizing antibody. Plasma samples were diluted 1:10 for all samples and 1:100 for a more dilute assay of seropositive samples at week3 and week 7 timepoints.

### SARS-CoV-2 protein and peptide microarray

Plasma samples were diluted 1:200 and used to probe a single SARS-CoV-2 protein and peptide microarray (CDI Labs). After probing arrays with serum antibodies, the arrays were washed, labeled with an Alexa647-anti-human IgG Fc secondary antibody and scanned using a GenePix 4000B scanner. Array data was collected using the MAGPIX software (Innopsys). Each protein or peptide was represented in triplicate on the microarray. There were positive control proteins (human IgG, anti-human IgG, and ACE2_Fc) and blank wells served as negative controls. The signal intensity was measured in the detection channel 635 nm (F635). The average of the F635 for each peptide was calculated and log2 transformed for graphing. *Z*-scores were calculated across all peptides for each individual prior to *t* test analysis with Bonferroni adjustment for multiple comparisons.

### SARS-CoV-2 antigen IgG subclass (IgG1, IgG2, IgG3, IgG4) binding

Antigens: SARS-CoV-2 spike protein RBD (Genscript # Z03483), SARS-CoV2 nucleocapsid protein (Genscript #Z03488), SARS-CoV-2 S1 subunit spike protein (Genscript #Z03501), SARS-CoV-2 S2 subunit spike protein (R&D Systems #10594-CV) were all diluted to 1 μg/mL in 0.1 M sodium bicarbonate and incubated on high-binding plates (corning #3369) overnight at 4 degrees. Serum was diluted to 1:30 in superblock buffer with sodium azide followed by subsequent 1:3 dilutions until a final dilution of 1:21870 was reached. Secondary antibodies were purchased from Southern Biotech: Mouse Anti-Human IgG1 Fc-HRP (#9054-05), Mouse Anti-Human IgG2 Fc-HRP (#9060-05), Mouse Anti-Human IgG3 Hinge-HRP (#9210-05), Mouse Anti-Human IgG4 Fc-HRP (#9200-05). Secondary antibody dilutions were done in superblock buffer without sodium azide within range of manufacturers recommendations: IgG1 1:6000, IgG2 1:5000, IgG3 1:7000, IgG4 1:6000. SureBlue Reserve Microwell Substrate (VWR #95059-294) was added to each well and incubated in the dark for 15 min. Absorbance was measured at 450 nm immediately after 0.33 N HCl Acid Stop solution was added to the plate.

### Statistical analysis

All statistical analyses were performed using Prism 8.0 (GraphPad Software Inc.) software and R. For group comparisons, an unpaired nonparametric Wilcoxon-Mann-Whitney test was used with a two-tailed *P* value reported. We used a *P* value significance threshold of *P* < 0.05. Both the Wilcoxon-Mann-Whitney and descriptive statistical analysis such as mean, median, and range were calculated using non-transformed data. For the peptide microarray data, the log2 fluorescence intensity was used for the heatmap and these values were transformed into *Z*-score based on row (sample) for the *Z*-score plotting of the group average. We selected *Z*-score values > 1 (one standard deviation above mean) to identify immunodominant peptides within the vaccine group.

## Results

### SARS-CoV-2 antibody responses after vaccination in seropositive and seronegative individuals

Peripheral blood was collected before vaccination at baseline (week 0), after primary immunization (week 3), and secondary booster immunization (week 7) from individuals who had no known history of infection (seronegative; *N* = 152) or laboratory-confirmed SARS-CoV-2 infection 30–60 days (seropositive; *N* = 42) prior to the administration of the BNT162b2 vaccine (administered at week 0 and week 3). This population consisted of mostly adult middle aged, white, females who did not identify as Hispanic or Latino (Additional file 1: Table S1).

**Fig. 1:**
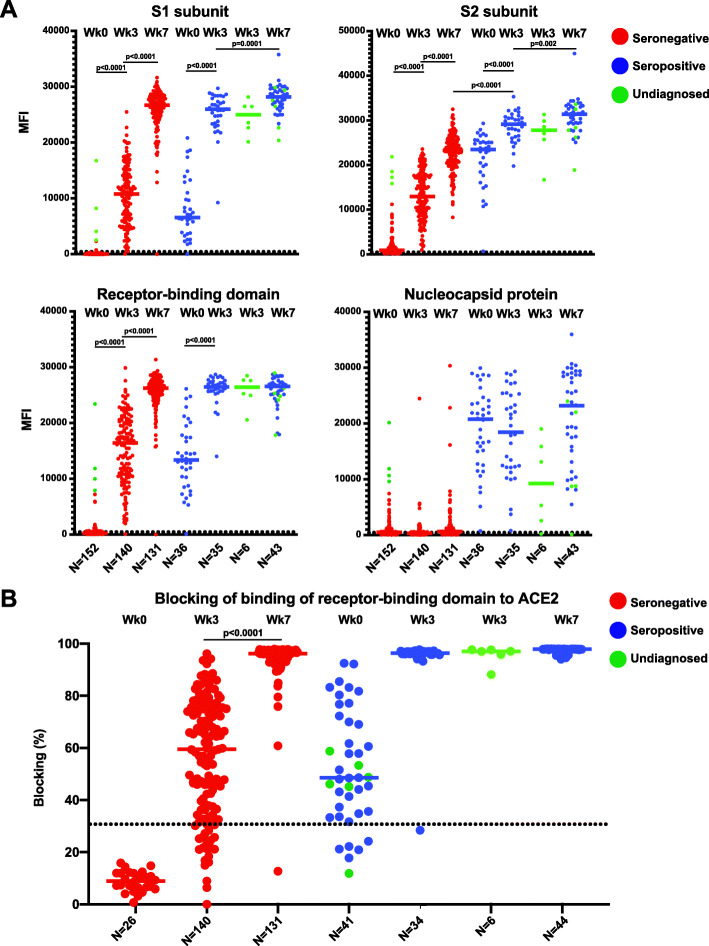
Antibody response to SARS-CoV-2 mRNA vaccine. **A** Multiplex bead-based antibody binding assay that measures the IgG antibody response to 4 SARS-CoV-2 viral antigens (S1, S2, RBD, and NP). Median Fluorescent Intensity (MFI) is shown and background well subtraction has been used to remove nonspecific signal. Each dot represents an individual at baseline before vaccine (week 0), 3 weeks after the first dose of vaccine (week 3) or 4 weeks after the second dose (week 7). Bars represent the group median. The number of individuals in each group are shown below the graphs. Individuals with a previous history of SARS-CoV-2 infection (seropositive; blue), no previous history of infection (seronegative; red), and individuals with possible undiagnosed infection (green). The dashed line indicates a threshold determined by the sum of the mean and standard deviation for the negative control (beads without antigen). **B** Neutralization antibody proxy assay that determines the level of antibodies that block the RBD-ACE2 receptor-binding expressed as the percentage of binding that was blocked relative to control with no plasma (representing maximum binding). The assay threshold for positivity was 30%. Each point represents an individual at baseline before the vaccine, 3 weeks after the first dose of vaccine (week 3) or 4 weeks after the second dose (week 7). Bars represent the group median. The number of individuals in each group are shown below the graphs. Individuals with a previous history of SARS-CoV-2 infection (seropositive; blue), no previous history of infection (seronegative; red), and individuals with possible undiagnosed infection (green). Statistical tests for significant differences between groups were unpaired, two-tailed Wilcoxon-Mann-Whitney test with a significant threshold of *P* < 0.05

We first measured immunoglobulin G (IgG) antibody levels at the three timepoints (week 0, week 3, and week 7) to SARS-CoV-2 spike subunit proteins (spike subunit 1 (S1), spike subunit 2 (S2), receptor-binding domain (RBD)) and nucleocapsid protein (NP) using a multiplexed bead-based binding assay. As expected at baseline, the seronegative group had low median fluorescence intensity (MFI) for all four antigens (S1, 16.8; S2, 886.7; RBD, 218.2; NP, 482.9) whereas the seropositive group had significantly higher median antibody binding levels (S1, 10793.3; S2, 12923; RBD, 16409.7; NP, 20766). Cross-reactivity of pre-existing antibody immunity has been reported in the absence of SARS-CoV-2 infection [20–24]. In parallel, we analyzed a group of individuals that had peripheral blood collected prior to the emergence of SARS-CoV-2 and the COVID-19 pandemic in the year 2019 (Additional file 1: Figure S1A). We found that the median MFI of the groups (S1, 20.5; S2, 732; RBD, 331.3; NP, 759.8) in the pre-pandemic individuals were similar to the baseline levels of the seronegative group prior to vaccination. Interestingly, the MFI for RBD was consistently higher than S1 in the prepandemic samples and further study into the protein background levels or conformation-dependent nature of the antibody response should be performed. Thus, MFI levels significantly above the seronegative individuals at baseline or the individuals collected prior to the pandemic would represent elevation in antibody levels due to vaccination or more recent SARS-CoV-2 infection. The lowest median MFI in the seronegative group was to the S1 protein, with a median MFI less than 20.5 for the seronegative vaccine group or group sampled prior to the COVID-19 pandemic (Additional file 1: Figure S1). We identified six individuals at baseline who had S1 antibody levels MFI ≥ 1000, similar to the levels observed at baseline in the seropositive group. These individuals also had higher levels of antibody binding to the other SARS-CoV-2 antigens tested and may have had undiagnosed or asymptomatic infection prior to vaccination (Additional file 1: Figure S1B). After primary immunization (week 3), both seronegative and seropositive individuals had significantly increased IgG antibody titers to all three spike subunit antigens when compared to baseline (S1, S2, RBD; *P* < 0.0001; Fig. 1A). Furthermore, seropositive individuals had significantly higher titers to all spike antigens when compared to the seronegative group at week 3 after primary immunization (*P* < 0.0001; Fig. 1A). The undiagnosed individuals resembled seropositive participants in week 3 serological assays and therefore we added them to the seropositive group for subsequent analysis at week 7 (green points). While the NP is contained in the whole virus, only the SARS-CoV-2 spike protein is a vaccine antigen. As expected, we found higher NP antibody titers only in the group with prior natural infection (seropositive), and there were no significant changes in NP antibody levels after vaccination in either group (seropositive or seronegative) (Fig. 1A). Four weeks after the second vaccine dose (week 7), seronegative individuals had significantly increased antibody titers to all 3 spike subunit proteins when compared to week 3 (*P* < 0.0001), whereas seropositive individuals had significant increases to S1 and S2 but not the RBD, with smaller magnitudes of increases compared to week 3 levels. Antibody titers in seropositive individuals at week 3 were comparable to week 7 titers in seronegative individuals for S1 and RBD; however, seropositive individuals had significantly higher S2 antibody levels at both week 3 and week 7 when compared to seronegative individuals (*P* < 0.0001; Fig. 1A).

We next used an assay that allows indirect detection of SARS-CoV-2 neutralizing antibodies through determination of antibody blocking of SARS-CoV-2 RBD binding to the human host viral receptor angiotensin-converting enzyme 2 (ACE2). This assay has been demonstrated to have a significant correlation with levels of neutralizing antibodies determined by primary virus or pseudovirus neutralization assays [25]. Levels of blocking antibodies were significantly increased after primary vaccination (week 3) for both seropositive and seronegative groups, and the seropositive group had significantly higher blocking antibodies when compared to the seronegative group (*P* < 0.0001; Fig. 1B). The second vaccine dose only significantly increased blocking for the seronegative group at week 7 (*P* < 0.0001; Fig. 1B). There was no significant difference in blocking antibodies in the seropositive group after the first vaccine dose (week 3) when compared to the seronegative group after the second vaccine dose (week 7). The median percent blocking for the seropositive samples was 96.3% at week 3 and 97.9% at week 7. We further performed an additional 10-fold dilution of the plasma samples in order to determine if there were any increases in the magnitude of neutralizing antibodies in seropositive individuals after the second vaccine dose. Using this plasma dilution, we found that there was an increase in the seropositive group from week 3 (median 89.4%) to week 7 (median 93.4%) that was modest in magnitude but statistically significant (*P* = 0.02; Additional file 1: Figure S2). The threshold for positive detection of SARS-CoV-2 neutralizing antibodies has been determined to be ≥ 30% for this assay. Using this threshold as a marker of seropositivity after vaccination, there were 86.1% and 97.5% of individuals who had antibody levels above the threshold after the first immunization in the seronegative and seropositive/undiagnosed groups, respectively (Fig. 1B). After the second dose, nearly all participants in both groups had positive neutralizing antibody titers (seronegative: 99.2%; seropositive: 100%; Fig. 1B). These results demonstrated that individuals with prior SARS-CoV-2 infection have higher magnitudes of binding and neutralizing antibodies after a single dose that is equivalent to levels observed in seronegative individuals after two vaccine doses. Moreover, seropositive individuals only receive a minor increase in antibody binding magnitude after the second dose.

### Impacts of sex and age on primary vaccine SARS-CoV-2 vaccine response

**Fig. 2:**
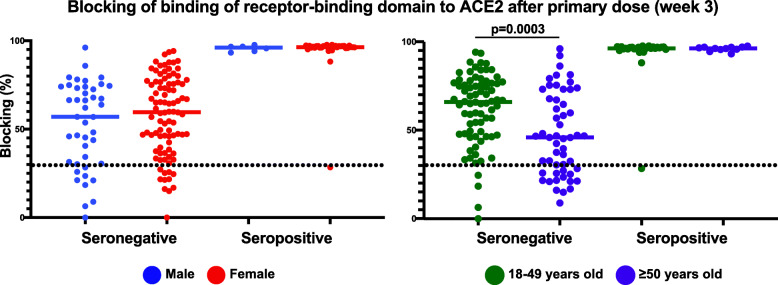
Antibody response to primary immunization based on sex and age. **A** Results of the neutralization proxy blocking assay after primary immunization at week 3 with individuals stratified by sex (male, female) or age (18–49 years old, ≥ 50 years old). Each point represents an individual 3 weeks after the first dose of vaccine (week 3). Bars represent the group median. Statistical tests for significant differences between groups were unpaired, two-tailed Wilcoxon-Mann-Whitney test with a significant threshold of *P* < 0.05

Next, we determined the contribution of sex and age to vaccine neutralizing antibody responses by stratifying the week 3 blocking assay results by sex and age. After the first vaccine dose (week 3), no significant differences in blocking antibody levels based on sex were detected (Fig. 2). However, we did observe that older individuals (≥ 50 years of age) in the seronegative group had significantly lower blocking antibodies compared to younger individuals (< 50 years of age) after primary immunization, but no significant difference in age was observed for the seropositive group (*P* = 0.0003; Fig. 2).

### SARS-CoV-2 IgM and IgA isotype response during vaccination

**Fig. 3:**
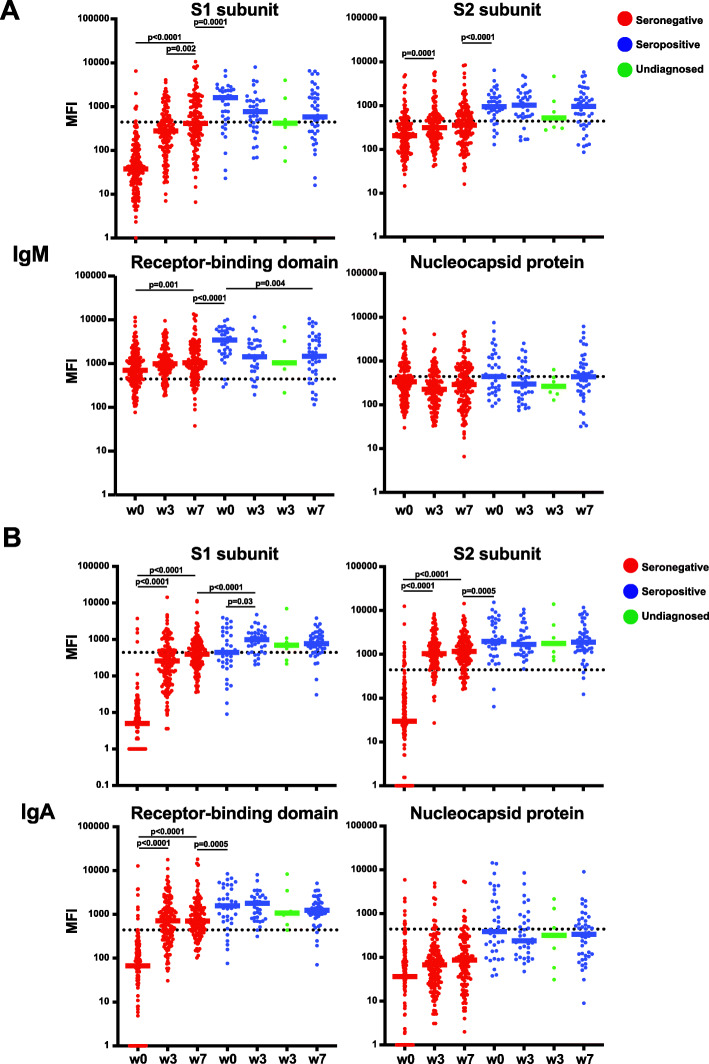
IgM and IgA antibody isotype responses to SARS-CoV-2 after immunization. **A**, **B** Multiplex bead-based antibody binding assay that measures the IgM (**A**) or IgA-specific (**B**) antibody response to 4 SARS-CoV-2 viral antigens (S1, S2, RBD, and NP). Median Fluorescent Intensity (MFI) is shown and background well subtraction has been used to remove the nonspecific signal. Each dot represents an individual at baseline before vaccine (week 0), 3 weeks after the first dose of BNT162b2 COVID-19 vaccine (week 3) or 4 weeks after the second dose of BNT162b2 COVID-19 vaccine (week 7). Bars represent the group median. The number of individuals in each group are shown below the graphs in Fig. 1. Individuals with a previous history of SARS-CoV-2 infection (seropositive; blue), no previous history of infection (seronegative; red), and individuals with possible undiagnosed infection (green). The dashed line indicates a threshold determined by the sum of the mean and standard deviation for the negative control (beads without antigen). Statistical tests for significant differences between groups were unpaired, two-tailed Wilcoxon-Mann-Whitney test with a significant threshold of *P* < 0.05

While IgG isotype antibodies are the major surrogates of vaccine and infection-mediated immunity, the kinetics and contributions of IgM and IgA isotypes are less clear. We found that IgM antibody responses to S1, S2, and RBD were only significantly boosted after immunization in the seronegative individuals, whereas the antibody levels in seropositive individuals were not boosted and even waned by week 7 (Fig. 3A). However, IgM levels at baseline in the seropositive individuals remained significantly higher than levels elicited by two doses of vaccine (week 7) in seronegative individuals (Fig. 3A). Similarly, IgA antibody levels were only increased by vaccination in seronegative individuals, with no significant change during vaccination in seropositive individuals (Fig. 3B). Only IgM S1 antibody levels had a significant increase in seronegative individuals after the second dose, indicating a limited contribution of secondary immunization to IgM and IgA antibody responses (Fig. 3A, B). There was no change in seronegative or seropositive individuals IgM or IgA titers against NP over the course of vaccination as expected (Fig. 3A, B). These results indicated that immunization with SARS-CoV-2 vaccines could elicit IgM and IgA isotype antibodies in seronegative individuals, but not for individuals with a previous history of infection.

### IgG subclass response to SARS-CoV-2

**Fig. 4:**
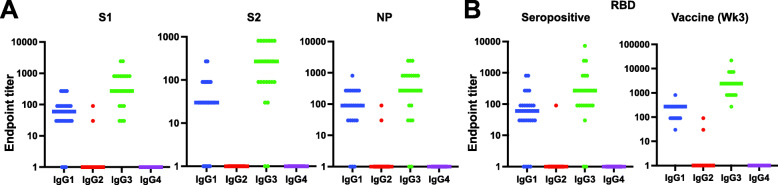
IgG subclass responses to SARS-CoV-2. **A**, **B** Dot plots of ELISA End Point titers calculated as the highest plasma dilution where the O.D. is 3× background. The bar represents the group median, and the points display individual data. Secondary antibodies specific for each IgG subclass (IgG1, IgG2, IgG3, IgG4) were used to determine subclass-specific responses. **A** Subclass responses to SARS-CoV-2 spike subunits (S1, S2) and nucleocapsid protein (NP) in SARS-CoV-2 infected individuals (*n* = 24) at baseline before the vaccine. **B** Subclass responses to SARS-CoV-2 receptor-binding domain (RBD) in SARS-CoV-2 infected individuals at baseline before vaccine (seropositive; *n* = 24) and three weeks after the first dose of BNT162b2 COVID-19 vaccine in seronegative individuals (vaccine; *n* = 24)

There are four IgG subclasses (IgG1, IgG2, IgG3, IgG4) that differ in efficiencies in recruiting immune effector cells [26, 27]. In a recent study, it was determined that SARS-CoV-2-infected individuals make IgG1 and IgG3 subclass antibody responses to the spike RBD with little contribution from IgG2 or IgG4 subclasses [28]. Using IgG subclass-specific enzyme-linked immunosorbent assays (ELISAs), we found that in 24 SARS-CoV-2-infected individuals at baseline before immunization, IgG1 and IgG3 were the dominant subclasses for spike protein S1, S2, and RBD subunits and the NP (Fig. 4A; Additional file 1: Figure S3). Only two individuals made low titer IgG2 responses, and no individuals had detectable IgG4 antibody responses (Fig. 4A; Additional file 1: Figure S3). Similarly, after primary immunization with SARS-CoV-2 vaccine, seronegative individuals displayed the same pattern of subclass response to the spike RBD when compared to seropositive individuals at baseline, with IgG1 and IgG3 levels being the highest. These results indicated that similar class-switch antibody responses to SARS-CoV-2 spike protein occurred after both vaccination and natural infection (Fig. 4B; Additional file 1: Figure S3).

### High-resolution antibody epitope determination by SARS-CoV-2 spike peptide microarray

**Fig. 5:**
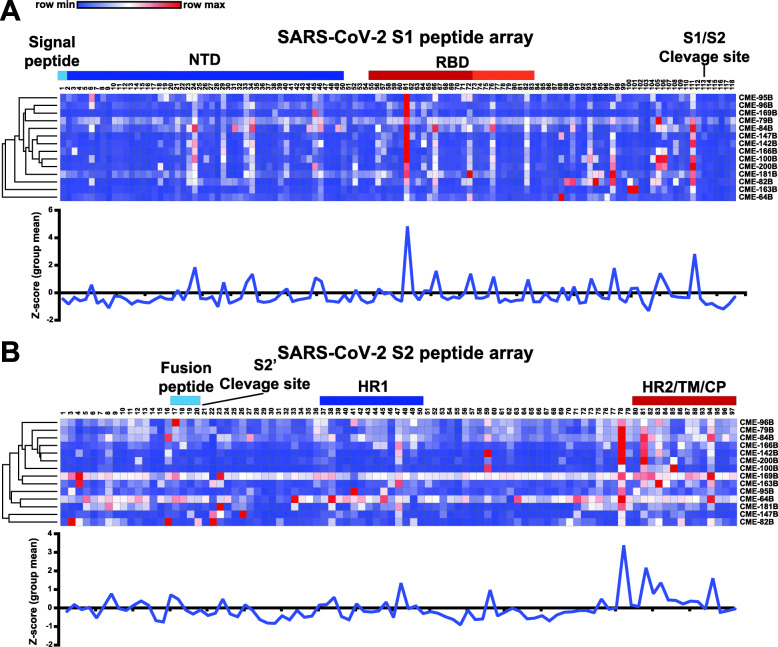
High-resolution mapping of SARS-CoV-2 antibody responses after COVID-19 vaccine. **A**, **B** Plasma IgG binding to SARS-CoV-2 peptides spanning the SARS-CoV-2 spike protein (**A** S1 and **B** S2 subunits separated;12-mer overlapping peptides) in 14 SARS-CoV-2 seronegative adults after primary COVID-19 immunization (week 3). Each peptide was printed in triplicate and the log2 of the average median fluorescent intensity (F635) for each peptide graphed. Row color corresponds to the minimum and maximum intensity for all peptides for each individual. Known regions of the spike protein are annotated above heatmaps. Orange section in RBD depicts critical ACE2 contact residues. Line graphs displaying the mean group Z-score for each peptide in S1 and S2 from the vaccine group (blue; *n* = 14)

Finally, we selected 14 seronegative individuals after primary immunization with the SARS-CoV-2 vaccine (week 3) and determined linear epitopes recognized by humoral immune responses using a SARS-CoV-2 spike peptide microarray that contained 196 soluble 12-mer overlapping peptides (Additional file 2). Heatmaps using the Log2 MFI values for each peptide were displayed for each spike subunit (Fig. 5; Additional file 3). Using a threshold of mean *Z*-score greater than 1 as “immunodominant,” we found that a peptide in the RBD (S1-61) was the most immunodominant in the S1 region, targeted by antibodies from most individuals tested (Fig. 5A; Additional file 4). There were also three other peptides in the RBD that had high antibody binding levels (S1–66, S1–72, S1–76), with only one occurring in the region identified to make critical contacts with host cell receptor ACE2 (S1-76; Fig. 5A). Outside of the RBD, there were also peptides highly recognized by antibodies within the N-terminal domain (NTD; S1–24, S1–34, S1–45) and near the S1/S2 cleavage site (S1–111, S1–105, S1–97; Fig. 5A). In the S2, the antibody response was mainly targeted to four peptides in the C-terminal region near the heptad repeat 2 (HR2), transmembrane, and cytoplasmic domains (S2–78, 21–81, S2–83, S2–94), but there was a single peptide in the heptad repeat 1 (HR1) domain that was also immunodominant (S2–47; Fig. 5B, Additional file 4). This data identified regions in the spike protein that are commonly recognized by antibodies from many SARS-CoV-2 vaccinated individuals and may reveal epitopes critical for vaccine-mediated protection.

## Discussion

We observed equivalent antibody levels after a single dose of BNT162b2 SARS-CoV-2 vaccine in previously infected SARS-CoV-2 seropositive individuals compared to seronegative individuals after two doses of vaccine. This observation is in line with several previous studies that showed after a single dose of SARS-CoV-2 mRNA vaccine, seropositive individuals had significantly higher titers when compared with seronegative individuals with no history of previous infection [16–19]. The data presented here imply that a second dose of vaccine in seropositive individuals does not significantly boost the antibody titers higher. This observation was also reported in another recent study [29]. One limitation of our study is that there may be smaller magnitude increases in antibody titers in the seropositive group after the second dose that is limited by our immunoassay detection platform. Improved assays with standard concentrations and dilution series along with further study of antibody functionality will be required to determine what are the benefits of repeated doses in both seropositive and seronegative individuals. Despite these observations, the impact of prior infection on other parameters of immunity, such as T cell-mediated immunity, as well as the duration of vaccine-mediated protection need further evaluation. Moreover, we only compared individuals that had prior infection 30–60 days before primary immunization to individuals with no history of infection, indicating that future studies are needed to determine if there are differences in antibody responses to vaccination in individuals who had prior infection beyond 60 days. Another limitation of our study is that there was an overrepresentation of women and individuals that identify as White compared to other ethnicities due to the demographics of our health care workers. Future studies will be required to efficiently determine the role of sex and ethnicity in the antibody response to SARS-CoV-2 vaccines. The lack of robust immune correlates of protection for the SARS-CoV-2 vaccines in humans impedes serial monitoring of protective immunity in these populations. Further definition of immune correlates of protection for these vaccines will allow potential alterations in vaccine regimens for specific individuals.

While IgG isotype antibodies are typically targeted for vaccines and therapeutics, IgA and IgM isotypes can play critical roles in protecting mucosal surfaces from pathogens. IgA antibodies have been shown to neutralize SARS-CoV-2 [30] and elevated levels of IgA in the blood are associated with influenza vaccine efficacy [31, 32]. We found that peripheral blood SARS-CoV-2 spike protein IgM and IgA responses were boosted after primary exposure in seronegative individuals but were not significantly increased in seropositive individuals during vaccination, or after the second dose of vaccine in seronegative individuals. This may indicate a peak response generated by primary exposure that is difficult to further boost in the blood by repeated exposure to antigen. Moreover, we further defined the contributions of the four IgG subclasses to SARS-CoV-2 binding and found that similar to natural infection, IgG1 and IgG3 dominated the response, with little contribution by IgG2 and IgG4 subclasses. Vaccine-elicited antibodies of different subclasses can differentially recruit and activate innate immune effector cells which express antibody receptors on their surface and this can significantly impact vaccine efficacy [33, 34]. During SARS-CoV-2 infection it has been shown that neutralizing antibodies rapidly develop and are predominantly IgG1 or IgG3 subclass [28]. These data demonstrated that vaccination could elicit similar antibody class-switching as observed during natural infection but the contributions to long-term immunity need further study.

Finally, we used a SARS-CoV-2 peptide array to identify immunodominant linear epitopes targeted by antibodies after vaccination. Studies from convalescent individuals with SARS-CoV-2 infection identified dominant neutralizing epitopes within the RBD as well as outside the RBD in the S1 and S2 regions of the spike protein [35, 36]. The two most immunodominant peptides were S1-61 within the receptor-binding domain and S2–78 at the C-terminal of the spike protein adjacent to the HR2. The peptide sequence of S1–61 is outside of the amino acid motif that contacts ACE2 so the significance of this epitope or its functional role is unclear and not described in previous studies. However, the peptide S2–78 has also been observed to be a region of immunodominance in antibody serology studies of natural SARS-CoV-2 infection [36–38]. Moreover, antibody response levels to this peptide have been shown to have a neutralizing activity and were associated with decreased COVID-19 disease severity [36, 37]. These observations raise the possibility that epitopes outside of the RBD that do not directly block the RBD-ACE2 interaction could be utilized to mitigate viral entry through other critical mechanisms. Similarly, we found that after primary immunization there are linear epitopes outside the RBD that are targeted by antibodies observed in many individuals. Future studies to determine if antibodies targeting these epitopes can neutralize the virus will unveil possible new viral mechanisms of action and targets for novel vaccine formulations.

## Conclusions

In conclusion, this study provides a high-resolution definition of the antibody responses to SARS-CoV-2 mRNA vaccination in individuals that had SARS-CoV-2 infection compared with individuals with no prior history of infection. Future studies of other immune parameters and the durability of immunity will be required to alter immunization regimens based on prior viral or vaccine exposure.

## Supplementary Information

**Additional file 1: Supplemental Table 1.** Study participant demographics. **Supplemental Figure 1.** Antibody binding to SARS-CoV-2 antigens at baseline in undiagnosed individuals. **Supplemental Figure 2.** Higher dilution of seropositive plasma samples after vaccine for neutralization assay. **Supplemental Figure 3.** IgG isotype responses to SARS-CoV-2.

**Additional file 2.** SARS-CoV-2 spike peptide sequences.

**Additional file 3.** Log2 Median fluorescence intensity values for each SARS-CoV-2 peptide.

**Additional file 4.** Z-scores for each SARS-CoV-2 peptide.

## Data Availability

Data that support the findings of this study are available from the corresponding author upon reasonable request.
