# Supplementary material for: Humoral immune responses during SARS-CoV-2 mRNA vaccine administration in seropositive and seronegative individuals
Source: BMC Med. 2021 Jul 26;19:169. doi: 10.1186/s12916-021-02055-9 (PMC8310732; doi:10.1186/s12916-021-02055-9)
Supplement: Supplementary file 1 — Additional file 1: Supplemental Table 1. Study participant demographics. Supplemental Figure 1. Antibody binding to SARS-CoV-2 antigens at baseline in undiagnosed individuals. Supplemental Figure 2. Higher dilution of seropositive plasma samples after vaccine for neutralization assay. Supplemental Figure 3. IgG isotype responses to SARS-CoV-2. [file 12916_2021_2055_MOESM1_ESM.pdf]

Additional File 1. Fraley et al. Humoral immune responses during SARS-CoV-2 mRNA vaccine administration in seropositive and seronegative individuals

Supplemental Table 1. Study participant demographics.
Supplemental Figure 1. Antibody binding to SARS-CoV-2 antigens at baseline in undiagnosed individuals.
Supplemental Figure 2. Higher dilution of seropositive plasma samples after vaccine for neutralization assay.
Supplemental Figure 3. IgG isotype responses to SARS-CoV-2.

Additional File 2. SARS-CoV-2 spike peptide sequences.
Additional File 3. Log2 Median fluorescence intensity values for each SARS-CoV-2 peptide.
Additional File 4. Z-scores for each SARS-CoV-2 peptide.

Table S1. Study participant demographics

	**Seropositive** (COVID19+; N=42)	**Seronegative** (COVID19-; N=152)
**Age**	Median 38 years old<br>Range 25-73 years old	Median 46 years old<br>Range 22-75 years old
**Gender**	Male: 5<br>Female: 37	Male: 46<br>Female: 105
**Race**	White: 39<br>Black or African American: 0<br>American Indian or Alaska Native: 1<br>Asian: 2<br>Native Hawaiian or Other Pacific Islander: 0<br>Multiracial: 0<br>Unknown: 0	White: 133<br>Black or African American: 3<br>American Indian or Alaska Native: 0<br>Asian: 7<br>Native Hawaiian or Other Pacific Islander: 0<br>Multiracial: 5<br>Unknown: 4
**Ethnicity**	Hispanic or Latino: 5<br>Not Hispanic or Latino: 36<br>Unknown: 1	Hispanic or Latino: 10<br>Not Hispanic or Latino: 129<br>Unknown: 13

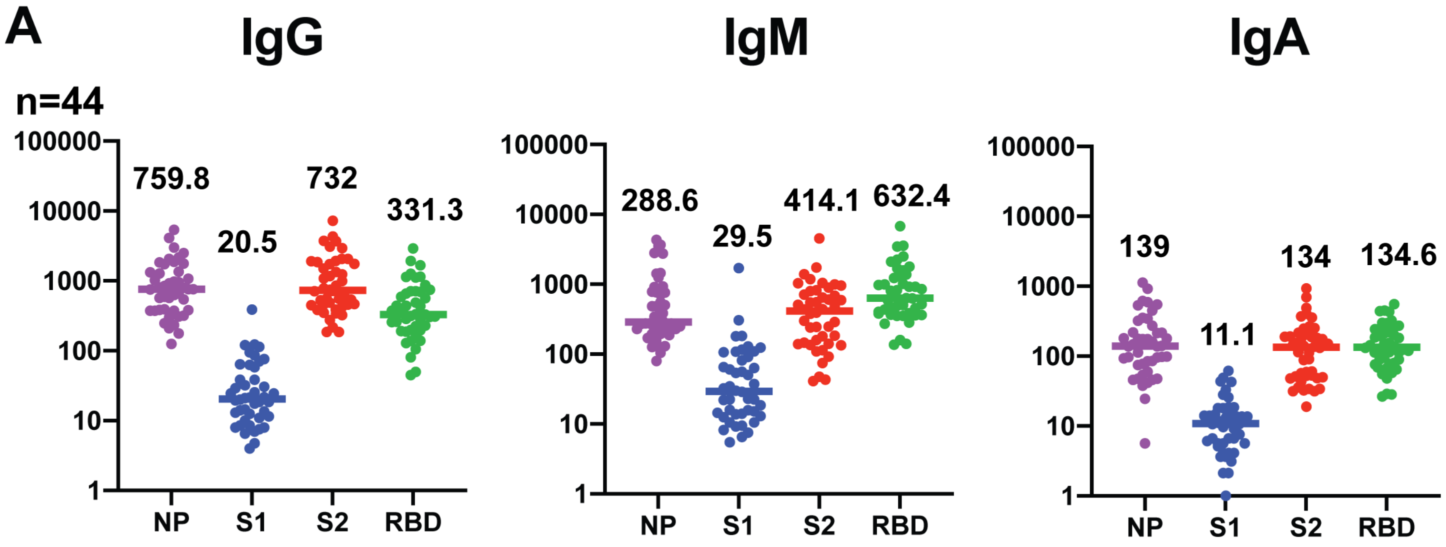

B

Original ID	S1	S2	RBD	NP
CME-131	26497.9	28374.6	25295	8793.2
CME-169	22661.4	18878.1	24132	128.2
CME-075	27573	27795	24792	8724
CME-081	29879.5	32502	28946.5	22007
CME-093	20391	26211	17823	23183
CME-118	29406	33599.5	26251	23974
Median	16.8	886.7	218.2	482.9

Supplemental Figure 1. Antibody binding to SARS-CoV-2 antigens at baseline in undiagnosed individuals. (A) Multiplex bead-based antibody binding assay that measures the IgG, IgM or IgA antibody isotype-specific responses to 4 SARS-CoV-2 viral antigens (S1, S2, RBD and NP). Median fluorescent Intensity (MFI) is shown and background well subtraction has been used to remove nonspecific signal. Each dot represents an individual (n=44) that had blood collected prior to the COVID-19 pandemic in 2019. Bars represent group median and median values displayed above groups. **(B)** Net MFI for the SARS-CoV-2 antigens (S1, S2, RBD, NP) for the six individuals identified that may have undiagnosed/asymptomatic prior infection at baseline. The median MFI for the seronegative group at baseline is shown at the bottom.

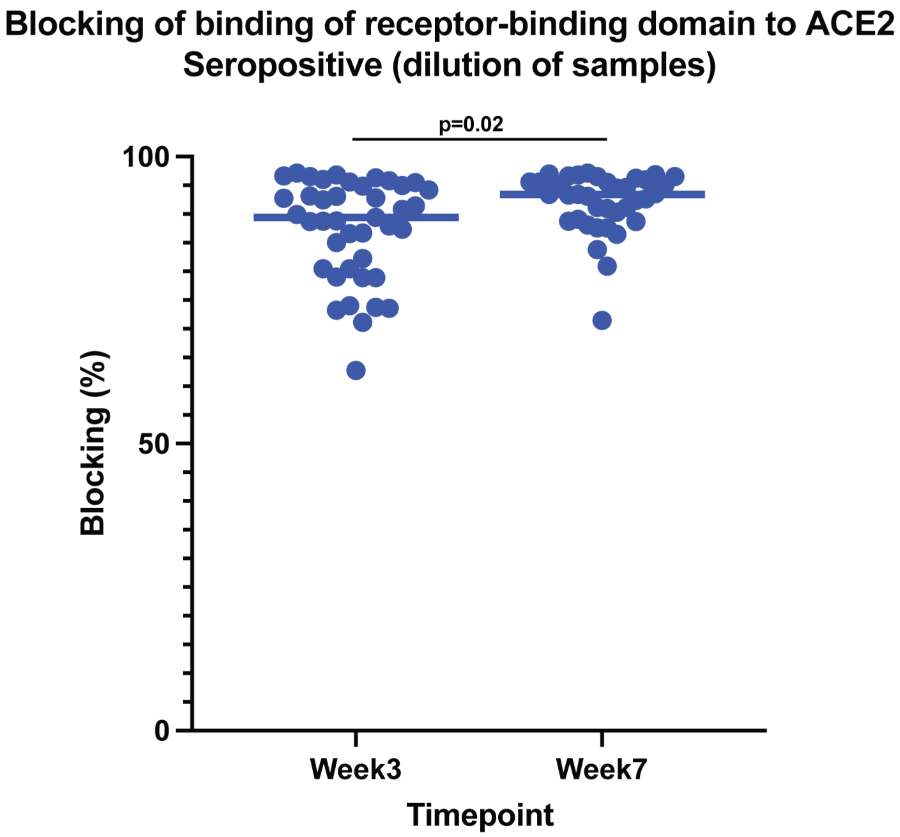

Supplemental Figure 2. Higher dilution of seropositive plasma samples after vaccine for neutralization assay. Neutralization antibody proxy assay that determines the level of antibodies that block the RBD-ACE2 receptor binding expressed as the percentage of binding that was blocked relative to control with no plasma (representing maximum binding). Plasma was diluted 1:100 for these samples. The assay threshold for positivity was 30%. Each point represents an individual at baseline before vaccine, 3 weeks after the first dose of vaccine (week 3) or 4 weeks after the second dose (week 7). Bars represent group median. Statistical tests for significant differences between groups were unpaired, two-tailed Wilcoxon-Mann-Whitney with a significant threshold of $P<0.05$.

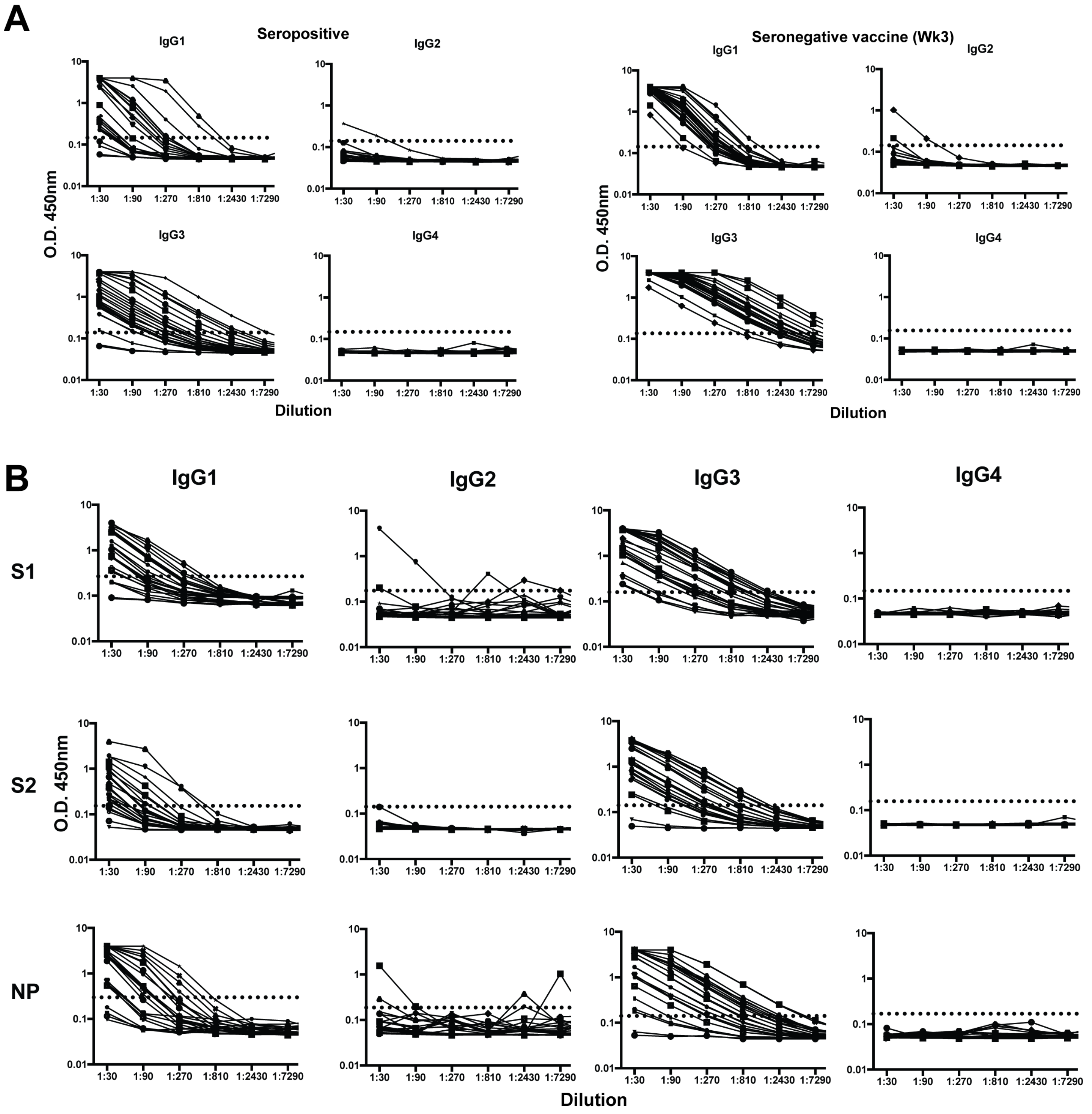

Supplemental Figure 3. IgG subclass responses to SARS-CoV-2. (A-B) O.D 450nm measurements after ELISA for each dilution for **(A)** RBD and **(B)** S1, S2 and NP proteins for each IgG subclass. Endpoint titers shown in Figure 4.
